# Endocrine, Metabolic, and Reproductive Responses to Bee Pollen Supplementation During Seasonal Anestrus in Goats in a Time-Dependent Manner

**DOI:** 10.3390/biology15201846

**Published:** 2026-10-15

**Authors:** Adriana Rojas, Reagan N. Sims, Brian L. Bruner, Adeyemi A. Olanrewaju, Jaime M. Cavazos-Galindo, Venancio Cuevas-Reyes, Morteza Hosseini Ghaffari, Cesar A. Rosales-Nieto

**Affiliations:** 1 Department of Agricultural Sciences, Texas State University, 601 University Dr, San Marcos, TX 78666, USA; 2 Medical Laboratory Science, Texas State University, San Marcos, TX 78666, USA; 3 Centro de Fomento Ganadero Vallecillo, Universidad Autónoma de Nuevo León, Vallecillo 65415, Nuevo León, Mexico; 4 Instituto Nacional de Investigaciones Forestales, Agrícolas y Pecuarias, Campo Experimental Valle de México, Texcoco 56250, Estado de Mexico, Mexico; 5 Research Institute for Farm Animal Biology (FBN), 18196 Dummerstorf, Germany

**Keywords:** bee products, energy metabolism, reproduction, endocrine response, SCFAs

## Abstract

Goats in subtropical and temperate regions experience seasonal anestrus during long-day conditions. This study evaluated the effects of bee pollen (BP) supplementation on circulating metabolic and endocrine measures, plasma short-chain fatty acids (SCFAs), and reproductive outcomes. Thirty crossbred Spanish × Boer does were assigned to T-CTL (n = 15) or T-BP (n = 15). After a 7-day adaptation period, T-BP received 10 g/day BP for 42 days (approximately 0.31 g/kg initial body weight/day), while T-CTL received carrier feed alone. We measured plasma glucose, insulin, insulin-like growth factor 1 (IGF-1), progesterone (P4), and SCFAs. BP supplementation did not significantly (*p* > 0.05) alter plasma glucose, insulin, IGF-1, or overall P4 concentrations. Only five does became pregnant (T-BP, 2/15; T-CTL, 3/15); therefore, fertility, prolificacy, and reproductive rate were summarized descriptively. Most plasma SCFAs did not show a consistent treatment-dependent temporal response. Isovalerate showed a significant treatment × date interaction, whereas hexanoate differed overall between treatments without a significant treatment × date interaction. Under this single-dose protocol, BP did not consistently modify circulating metabolic–endocrine markers or improve reproductive performance during the suboptimal breeding season. Future studies should include dose-ranging, direct rumen measurements, and adequately powered reproductive endpoints.

## Simple Summary

Goats in subtropical and temperate regions commonly undergo seasonal anestrus. This study evaluated whether bee pollen (BP) supplementation influenced circulating metabolic and endocrine measures, plasma short-chain fatty acids (SCFAs), and reproductive outcomes. Thirty Spanish × Boer does received carrier feed alone or 10 g/day BP for 42 days after a 7-day adaptation period. Plasma glucose, insulin, insulin-like growth factor 1 (IGF-1), progesterone (P4), and SCFAs were measured. BP did not significantly alter glucose, insulin, IGF-1, or overall P4 concentrations. Only five does became pregnant, so fertility, prolificacy, and reproductive rate were summarized descriptively and did not indicate improved reproductive performance. Most plasma SCFAs did not show a consistent treatment-dependent temporal response; isolated date-specific contrasts were considered exploratory when the treatment × date interaction was nonsignificant. Under the conditions of this study, 10 g/day BP did not overcome seasonal reproductive inhibition or produce consistent endocrine–metabolic effects.

## 1. Introduction

Reproductive efficiency in small ruminants from subtropical and temperate zones is significantly limited by seasonal anestrus, a phenomenon mainly regulated by photoperiod and metabolic status. During the spring and summer, the suboptimal breeding season, longer day lengths suppress melatonin secretion, reduce gonadotropin release, and reduce ovarian activity [1,2,3,4]. This cessation of sexual activity limits annual production efficiency in goat herds and requires effective management strategies. Although photoperiod is the primary environmental cue, metabolic signals also play a key role in modulating the hypothalamic–pituitary–gonadal (HPG) axis. Urrutia-Morales et al. [3] demonstrated this in goats at a subtropical location, where increased body weight shortens the anoestrus period. Furthermore, hormonal exogenous interventions, such as progestogens and melatonin implants, have been used to reactivate ovarian activity, but the results remain inconsistent [5,6]. Therefore, nutritional strategies that can manipulate neuroendocrine signals to overcome seasonal inhibition without the variability associated with hormonal treatments remain needed.

Beyond direct endocrine intervention, metabolic status centrally regulates reproductive competence. Short-chain fatty acids (acetate, propionate, and butyrate) serve not only as essential energy substrates for ruminants but also as metabolic regulators influencing hepatic glucose production and insulin response [7]. Elevated propionate levels, for example, have been positively associated with progesterone (P4) secretion by mediating insulin sensitivity, suggesting an indirect link between microbial fermentation products and reproductive hormone synthesis [8]. Furthermore, systemic metabolites like glucose and key growth factors, particularly insulin and insulin-like growth factor 1 (IGF-1), directly influence ovarian function [9]. Insulin and IGF-1 stimulate granulosa cell proliferation and steroidogenesis, promoting P4 production and optimizing ovarian receptivity [10]. Therefore, nutritional approaches that enhance energy availability and upregulate these growth factors may support reproduction during periods of energy imbalance or seasonal inactivity [11].

Bee pollen (BP) is generally described as a nutrient-dense natural product containing proteins, essential amino acids, polyunsaturated fatty acids, and bioactive compounds such as polyphenols and flavonoids [12]. These characteristics have been associated in the literature with antioxidant, anti-inflammatory, and immunomodulatory effects and with changes in energy metabolism and reproductive function in monogastric species [13,14,15]. Tryptophan in BP has also been discussed as a potential precursor of serotonin and melatonin [16]. In small ruminants, melatonin is a key mediator of photoperiodic responses [17]. However, BP composition varies with botanical and geographic origin [18], and the botanical source and total phenolic/flavonoid content of the BP used in the present study were not determined; therefore, these constituents are discussed as general characteristics of BP rather than experimentally quantified properties of the supplement used here.

Despite reported benefits in other species, it remains unknown whether BP supplementation is sufficient to support ovarian activity in goats during the suboptimal breeding season. Current literature lacks comprehensive evaluations of how BP affects the interplay among circulating fermentation-related metabolites, metabolic hormones, and reproductive outcomes in small ruminants. Tryptophan is commonly reported as a constituent of BP and provides a biological rationale for potential effects on melatonin synthesis [16], while melatonin is a key mediator of photoperiodic responses in small ruminants [17]. However, the botanical source and detailed bioactive composition of the BP used here were not characterized, and melatonin was not measured. These considerations support evaluating BP as a non-hormonal nutritional approach to seasonal reproduction while requiring cautious mechanistic interpretation.

To address this knowledge gap, this study evaluated whether BP supplementation influenced circulating metabolic and endocrine markers, plasma SCFAs, and reproductive outcomes during the suboptimal breeding season. We hypothesized that BP supplementation would influence circulating metabolic and endocrine markers and reproductive outcomes during this period. The specific objectives were to assess the effects of BP on circulating glucose, insulin, IGF-1, and P4; characterize plasma SCFA profiles; and describe reproductive outcomes during exposure to fertile bucks.

## 2. Materials and Methods

The Institutional Animal Care and Use Committee of Texas State University approved the animal procedures (IACUC #9157), and reporting followed the ARRIVE guidelines [19].

The study was conducted at the Goat Unit of Texas State University, located at Freeman Ranch (29°56′10.79″ N, 98°00′18.00″ W), where summers are hot and winters are mild. Precipitation varies throughout the year, with May typically receiving the most rainfall. During the study period (May–July), temperatures generally ranged from 31 °C to 39 °C. Temperatures, precipitation, and humidity were above average during this period. Rainfall during the study period was 181.6 mm, with average temperatures of 31.4 °C in May and 38.9 °C in July. The recorded humidity was approximately 79%, and the calculated temperature-humidity index was 77.4. The environmental temperature during the experiment was generally 1 to 2 °C above normal compared with previous years [20].

### 2.1. Experimental Design

**Figure 1:**
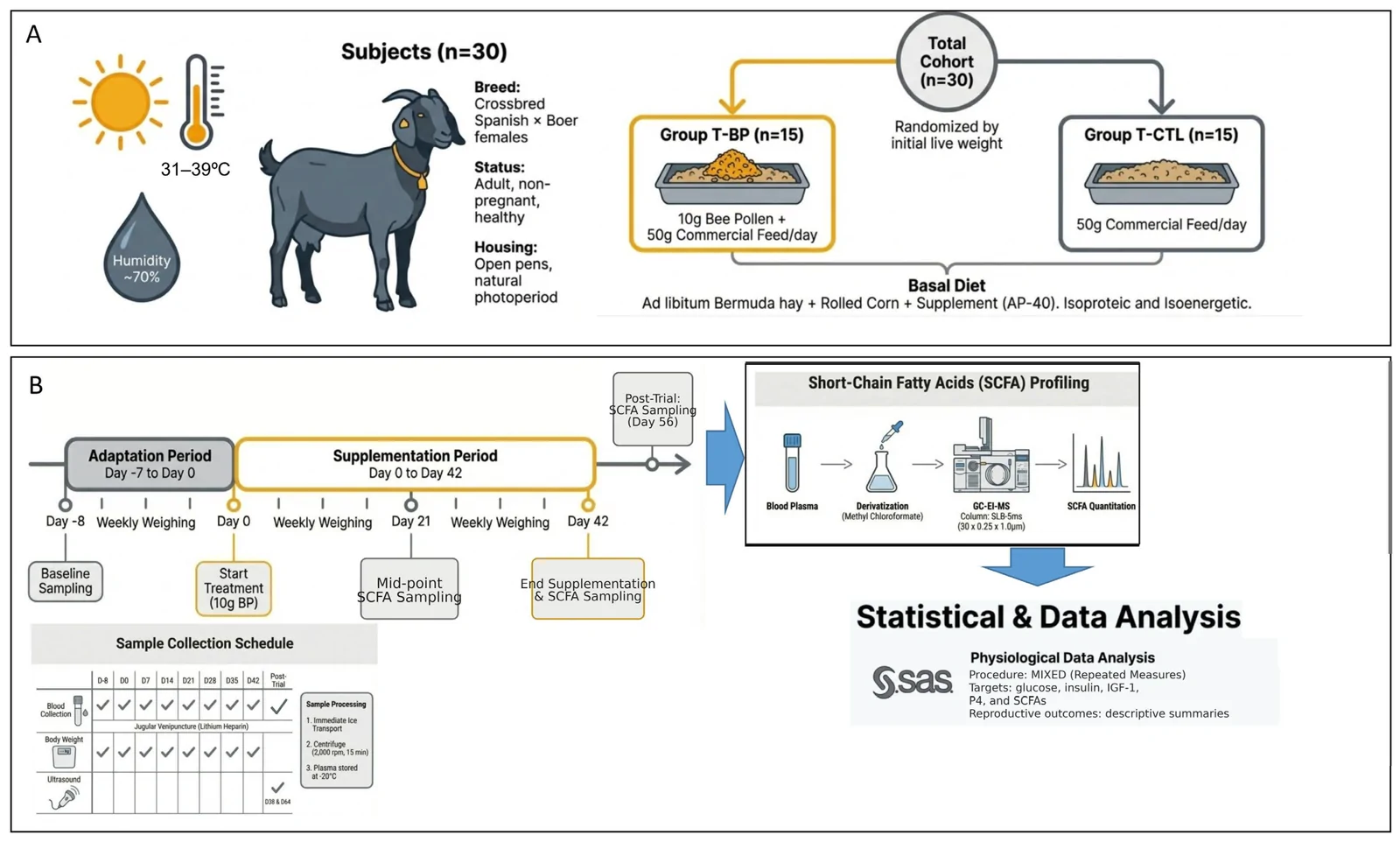
Experimental design and workflow for BP supplementation in crossbred goats. (**A**) Cohort, housing, and dietary regimen during the suboptimal breeding season. T-BP received 10 g BP/day, while T-CTL received carrier feed alone; both groups received the same basal maintenance diet. (**B**) Timeline showing the 7-day adaptation period, 42-day supplementation period, weekly live-weight and blood sampling, plasma SCFA sampling on days −8, 21, 42, and 56, and ultrasonography on days 38 and 64 after buck exposure. Fertile bucks were present from day 0 through day 42. Physiological repeated-measures outcomes were analyzed in SAS (Version 9.4), whereas reproductive outcomes were summarized descriptively. Metabolomic profiles from the same cohort were published separately in the companion study [21]. The schematic layout for this figure was initially drafted using Google Gemini (https://gemini.google.com/app, accessed on 13 November 2025) and subsequently refined, labeled, and verified by the authors for scientific accuracy.

To assess the effects of bee pollen (BP) supplementation on circulating glucose, insulin, insulin-like growth factor 1 (IGF-1), short-chain fatty acids (SCFAs), progesterone (P4), and reproductive outcomes, we enrolled 30 two-year-old crossbred Spanish × Boer does. The study used a balanced allocation of 15 animals per treatment group. We ranked goats by initial body weight and randomly assigned them to the BP-supplemented (T-BP) or control (T-CTL) group to achieve similar body-weight distributions. T-BP received a fixed oral dose of 10 g BP mixed with 50 g commercial balanced feed (Producer’s Pride^®^ with 12% CP and 2.96 MJ/kg ME), and T-CTL received the carrier feed without BP. The 49-day protocol comprised a 7-day adaptation period followed by 42 days of supplementation. We fed goats individually to ensure complete BP intake. We obtained BP from Walker Honey Farm; proximate composition was reported previously [21], but we did not determine the botanical source or total phenolic and flavonoid content. We did not perform a pilot dose-titration study; we selected the fixed dose from a prior ruminant study [22]. Based on a mean initial body weight of approximately 32.5 kg (T-BP: 32.2 ± 1.05; T-CTL: 32.9 ± 1.40), the dose was approximately 0.31 g/kg body weight/day and was not individually weight-standardized. All goats also received the same basal maintenance diet. The experimental design and sampling schedule are summarized in Figure 1. Plasma metabolomic data generated from the same cohort were published separately in the companion study [21] and are not reanalyzed, re-presented, or used for inference in the present manuscript.

### 2.2. Nutritional Diet

A total mixed ration was offered in feeders providing 30 cm of linear space per goat to minimize competition and ensure unrestricted access. Both groups received the same basal maintenance ration based on Bermuda hay, rolled corn, and commercial supplement (Table 1). SDK Laboratories assessed the nutritional composition of diets, including dry matter, crude protein, and metabolizable energy (Table 1). T-BP additionally received 10 g/day BP, whereas T-CTL received carrier feed alone; because the basal ration was not adjusted to compensate for BP nutrients, the complete treatment diets were not strictly isoproteic or isoenergetic. All feed was consumed, and no residual feed remained. Individual intake was not assessed. We included only clinically healthy animals, and all goats had continuous access to clean water and mineral and salt blocks. The diet was designed to reflect conditions of South Texas and comparable regions, characterized by variable rainfall and sparse vegetation [23].

### 2.3. Measurements

#### 2.3.1. Body Weight

The body weight was previously reported [21]. In brief, to assess the effect of BP on live weight gains, we recorded live weights weekly during the trial (Figure 1) using a mobile scale with a 1000 kg capacity and 1 kg precision (W-W Paul Scales, model 58SX; Duncan, OK, USA).

#### 2.3.2. Energy Metabolism Analysis

We collected plasma samples at multiple time points throughout the study to capture baseline and treatment-phase physiological responses (Figure 1). We collected blood samples weekly via jugular venipuncture using lithium heparin tubes (BD Vacutainer^®^, Preanalytical Solutions, Franklin Lakes, NJ, USA). Animals were acclimated to handling procedures to minimize stress-induced variability in metabolic parameters. After collection, we immediately placed blood samples on ice and transported them to the laboratory, where we centrifuged them at 2000× *g* for 15 min. We separated plasma and stored it at −20 °C until further analysis.

To evaluate energy metabolism, we measured plasma glucose, insulin, and IGF-1 concentrations as key indicators. The manufacturer validated the kits for caprine plasma (MyBioSource, San Diego, CA, USA). We performed all assays in duplicate and followed the manufacturers’ instructions. Quality control procedures included internal standards and plate-level controls to ensure assay accuracy and reproducibility. Glucose was measured using a glucose oxidase-based colourimetric assay (Catalogue #MBS169259), with absorbance recorded at 540 nm. The kit had a detection range of 0–100 µM and a sensitivity of 6.25 µM, as specified in the manufacturer’s documentation. Insulin concentrations were determined using a species-specific ELISA kit (Catalog #MBS947185). The kit had a detection range of 10 to 160 µIU/mL and a sensitivity of 2 µIU/mL; intra- and inter-assay CVs were <10% and <12%, respectively. IGF-1 levels were quantified using a caprine-specific ELISA kit (Catalog #MBS705626). The kit had a detection range of 62.5–1000 ng/mL and a sensitivity of 12.5 ng/mL, with intra- and inter-assay CVs reported at <8% and <10%, respectively.

SCFAs were quantified from plasma samples [25] at four time points using GC-MS method (Creative-Proteomics, Shirley, New York, USA) following validated protocols and quality assurance standards, with robust linear calibration. Free short-chain fatty acids were derivatized with methyl chloroformate in 1-propanol to yield propyl esters, followed by liquid–liquid extraction into hexane and analysis on an SLB-5 ms (30 × 0.25 × 1.0 μm) column with detection by GC-EI-MS in positive SIM mode. Deuterium-labeled fatty acids for each chain length served as internal standards, and calibration was performed using external six-point calibration curves for each SCFA. Plasma SCFAs were evaluated as circulating metabolites potentially associated with microbial fermentation and host energy metabolism.

#### 2.3.3. Reproductive Efficiency

Breeding was conducted during the suboptimal breeding season in the Northern Hemisphere. Bucks remain sexually active during this period [26], whereas female reproductive activity is seasonally reduced [27]. Fertility was defined as the percentage of exposed does confirmed pregnant. Prolificacy was defined as the number of fetuses per pregnant doe. We defined reproductive rate as the number of fetuses per 100 does exposed to fertile bucks. We exposed does to fertile bucks fitted with crayon harnesses (MatingMark; Hamilton, New Zealand), rotated bucks between groups every 12 h, and removed them after 42 d (two full reproductive cycles). Crayon markings were used to monitor behavioral estrus. Pregnancy and fetal number were assessed by transabdominal ultrasonography on days 38 and 64 after buck exposure.

Plasma P4 concentrations were measured at eight time points and treated as a continuous endocrine variable; no P4-based threshold or temporal criterion was used to classify ovulation or luteal activity. Blood samples were collected weekly via jugular venipuncture using lithium heparin tubes (BD Vacutainer^®^, Preanalytical Solutions, Franklin Lakes, NJ, USA), processed and stored as described above. Plasma P4 concentrations were measured using a commercial enzyme-linked immunosorbent assay (Catalog #MBS2700150) specific for caprine plasma according to the manufacturer’s instructions, with absorbance measured at 450 nm. The kit had a detection range of 3–200 ng/mL and a sensitivity of 0.78 ng/mL, as specified by the manufacturer. Samples were analyzed in duplicate, and standard controls were included on each plate.

#### 2.3.4. Statistical Analysis

We performed all statistical analyses using SAS [28]. We assessed data normality with PROC UNIVARIATE. We considered each goat an independent experimental unit. Plasma concentrations of SCFAs, glucose, insulin, IGF-1, and P4 were analyzed using the MIXED procedure by date and for repeated measures to account for time-dependent sampling within individual animals. We included treatment group as a fixed effect, sampling date as a repeated measure, and animal ID as a random effect to account for within-subject variability. We included body weight recorded on the sampling date as a covariate. The model assessed the main effects of treatment and time, as well as their interaction.

Data are presented as mean ± standard error of the mean (SEM). We determined significance at *p* ≤ 0.05, with tendencies assessed for *p* > 0.05 to *p* ≤ 0.10. The archived analysis record did not retain the covariance-structure selection criterion or documentation of a multiplicity-adjusted LSMEANS/SLICE procedure; these details could not be reconstructed retrospectively. Accordingly, isolated date-specific contrasts were not interpreted as treatment-dependent effects when the treatment × sampling-date interaction was nonsignificant.

We calculated reproductive outcomes from pregnancy and kids-born count data. Given the small number of pregnancies observed, fertility, prolificacy, and reproductive rate were summarized descriptively and no inferential statistical tests were performed for these outcomes.

## 3. Results

### 3.1. Energy Metabolism

#### 3.1.1. Impact of BP on Glucose Concentrations

**Figure 2:**
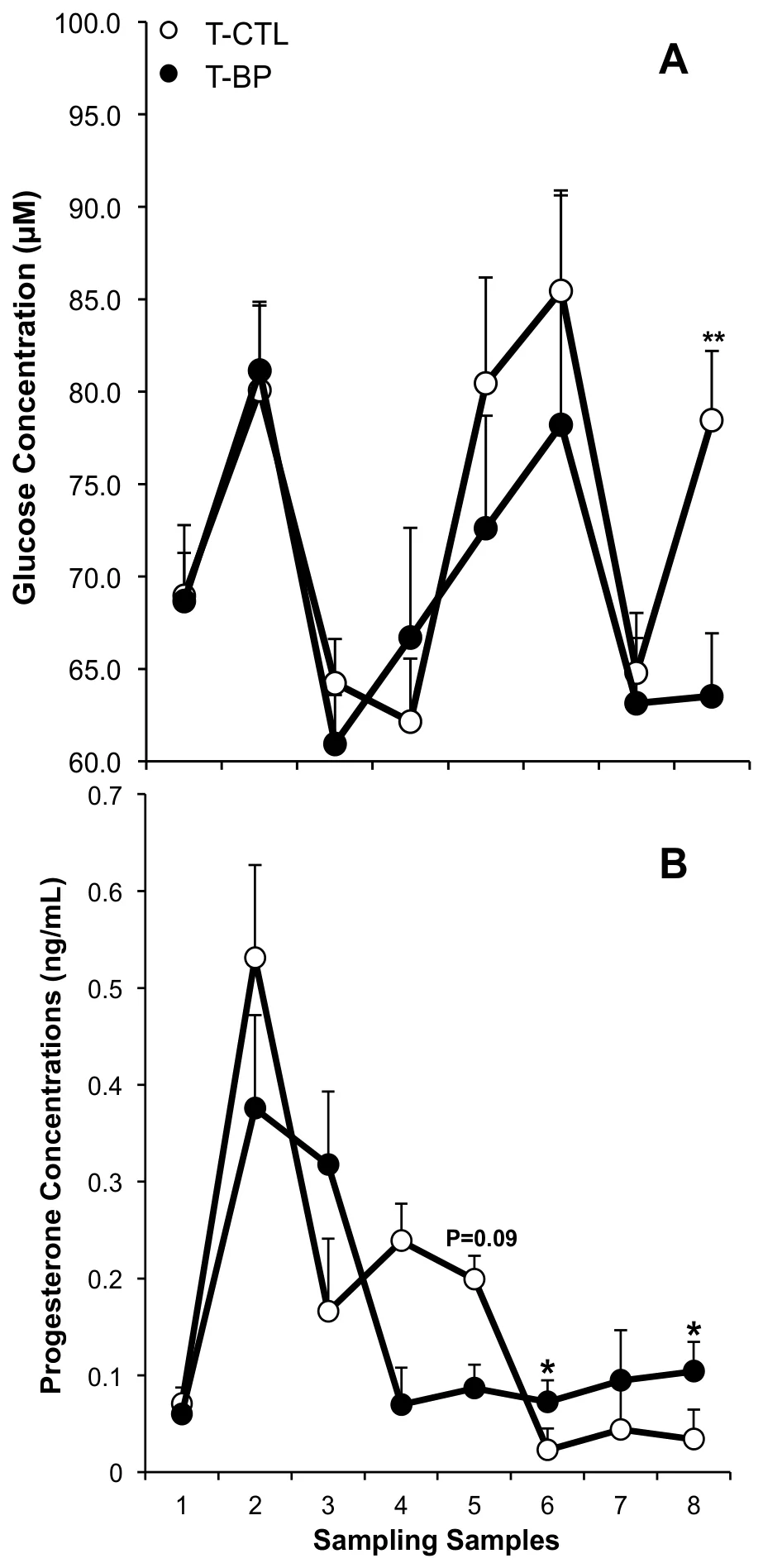
Mean (± SEM) circulating (**A**) glucose and (**B**) progesterone values in crossbred goats supplemented with 10 g/day BP (T-BP; dark circles) or carrier feed alone (T-CTL; white circles) during the suboptimal breeding season in a subtropical location (summer, Northern Hemisphere). Sampling date 1 is baseline (day −8), date 2 is day 0 and introduction of fertile bucks, date 5 is day 21, and date 8 is day 42. ** *p* < 0.01; * *p* < 0.05. For glucose, date-specific contrasts are considered exploratory because the treatment × date interaction was nonsignificant.

Blood glucose concentrations did not differ overall between treatments (*p* > 0.05), whereas concentrations varied among sampling dates (*p* < 0.001). The treatment × date interaction was nonsignificant (*p* > 0.05); therefore, the date-specific contrasts shown in Figure 2A are exploratory and not interpreted as treatment-dependent effects. Body weight did not influence glucose concentrations (*p* > 0.05).

#### 3.1.2. Impact of BP on Insulin and IGF-1 Concentrations

Table 2 shows mean circulating insulin and IGF-1 concentrations. Mean (±SEM) insulin and IGF-1 concentrations did not differ between treatments (*p* > 0.05; Table 2). Body weight did not affect insulin or IGF-1 concentrations (*p* > 0.05). Interaction between treatment and sampling date was not significant for either hormone (*p* > 0.05).

#### 3.1.3. Impact of BP on Short-Chain Fatty Acids

**Figure 3:**
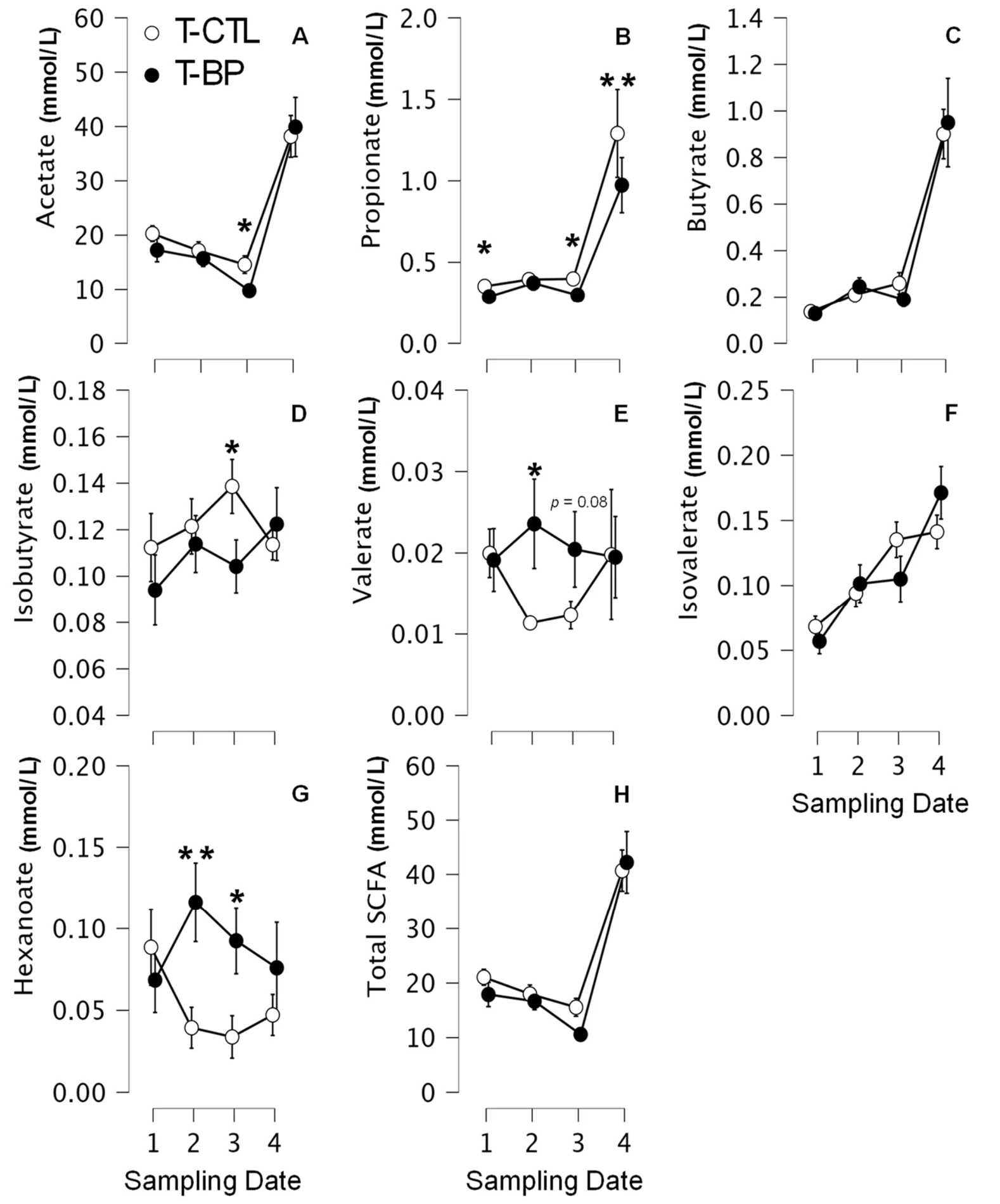
Mean concentrations of (**A**) acetate, (**B**) propionate, (**C**) butyrate, (**D**) isobutyrate, (**E**) valerate, (**F**) isovalerate, (**G**) hexanoate, and (**H**) total SCFAs in goats supplemented with 10 g/day BP (T-BP; dark circles) or carrier feed alone (T-CTL; white circles). Sampling dates represent day −8 (baseline; sampling date 1), day 21 (sampling date 2), day 42 (end of supplementation; sampling date 3), and day 56 (post-supplementation; sampling date 4). * *p* < 0.05; ** *p* < 0.01. Asterisks denote the originally reported single-date comparisons. When the overall treatment × date interaction was nonsignificant, we treated these contrasts as exploratory and did not interpret them as evidence of a treatment-dependent temporal response.

Mean acetate concentrations changed over time (*p* < 0.001; Figure 3A). The overall treatment effect and treatment × date interaction were nonsignificant; therefore, the isolated third-date contrast is exploratory and not interpreted as evidence of a treatment-dependent temporal response (Figure 3A). Body weight did not influence acetate concentrations (*p* > 0.05).

Mean propionate concentrations changed over time (*p* < 0.001; Figure 3B). The overall treatment effect and treatment × date interaction were nonsignificant; therefore, we treated isolated date-specific contrasts as exploratory and did not interpret them as evidence of a treatment-dependent temporal response (Figure 3B).

Mean butyrate concentrations increased over time (*p* < 0.001; Figure 3C) but did not differ between treatments within sampling dates (*p* > 0.05). Body weight did not influence butyrate concentrations (*p* > 0.05).

Mean isobutyrate and valerate concentrations did not differ overall between treatments, and their treatment × date interactions were nonsignificant (*p* > 0.05; Figure 3D,E). Isolated date-specific contrasts are therefore treated as exploratory and are not interpreted as treatment-dependent temporal responses. Body weight did not influence isobutyrate and valerate (*p* > 0.05).

Mean isovalerate concentrations increased and differed between treatments (*p* < 0.01; Figure 3F) and across time (*p* < 0.001). Body weight did not influence isovalerate concentrations (*p* > 0.05). The treatment and date interaction was significant (*p* < 0.05).

Mean hexanoate concentrations differed overall between treatments (*p* < 0.01; Figure 3G), while the treatment × date interaction was nonsignificant (*p* > 0.05). Accordingly, we did not interpret isolated date-specific contrasts as treatment-dependent temporal responses.

Total mean SCFA concentrations increased over time (*p* < 0.001; Figure 3H) but did not differ between treatments within sampling dates (*p* > 0.05). The treatment × date interaction was nonsignificant (*p* > 0.05). Body weight did not influence total SCFA concentrations (*p* > 0.05).

### 3.2. Impact of BP on Progesterone and Reproductive Outcomes

No significant treatment difference in P4 concentrations was observed (*p* > 0.05; Figure 2B). P4 varied over time (*p* < 0.001), and the treatment-by-date interaction was significant (*p* < 0.01).

Table 3 presents reproductive outcomes during the suboptimal breeding season. Fertility was 13% in T-BP (2/15) and 20% in T-CTL (3/15). Among pregnant does, one of two T-BP does carried twins, whereas none of the three T-CTL does did. Prolificacy was 1.5 fetuses per pregnant doe in T-BP (3 fetuses/2 pregnant does) and 1.0 in T-CTL (3/3). Reproductive rate was 20% in both groups (3 fetuses/15 exposed does).

## 4. Discussion

This study evaluated whether 10 g/day BP supplementation influenced circulating energy-related and endocrine markers, plasma SCFAs, and reproductive outcomes in female goats during the suboptimal breeding season. BP did not produce consistent treatment effects on glucose, insulin, IGF-1, or overall P4 concentrations, and only five does became pregnant. Most plasma SCFAs varied primarily over time rather than showing a consistent treatment-dependent temporal response. Overall, the findings do not support the conclusion that this single BP dose was sufficient to overcome seasonal reproductive inhibition under the conditions studied.

### 4.1. Effect of BP on Metabolism

Our results indicated that BP supplementation did not significantly alter circulating glucose concentrations. Although glucose concentrations varied across sampling dates, the treatment × date interaction was nonsignificant, and insulin sensitivity was not directly assessed. Taken together, these findings indicate no consistent treatment effect on circulating glucose at the oral dose administered. These findings contrast with studies in rodents and poultry, where BP improved glycemic control and enhanced energy metabolism [13,14,15]. Nevertheless, BP’s effect on glucose metabolism remains inconsistent across species. For instance, in rabbits, Sierra-Galicia et al. [29] reported enhanced glucose utilization following BP supplementation, while Abdel-Hamid and El-Tarabany [30] observed a reduction in blood glucose concentrations under similar conditions. In pre-ruminants, BP supplementation did not affect glucose concentrations [31]. The lack of a positive effect on glucose following BP supplementation warrants further investigation, as the differential response in ruminants may result from species-specific metabolite regulation and metabolism.

Similarly, insulin and IGF-1 concentrations did not differ significantly between groups; therefore, the study detected no treatment effect for these circulating hormones. Both hormones are responsive to nutritional and physiological status [32,33,34], and studies in monogastric species have reported variable responses to BP supplementation. For example, Abdel-Hamid and El-Tarabany [30] reported increased insulin and IGF-1 concentrations in rabbits supplemented with BP. Because the present study did not include a dose–response experiment, the data cannot determine whether a different BP dose would alter these endocrine endpoints.

### 4.2. Effect of BP on SCFAs

Previous studies have not documented the effects of BP on SCFAs in ruminants. In this study, plasma SCFAs changed over time, while several originally reported treatment differences were limited to individual dates despite nonsignificant treatment × date interactions. Those isolated contrasts are exploratory and are not interpreted as evidence of treatment-dependent temporal responses. Because rumen microbial composition and fermentation flux were not measured directly, the results do not establish that BP optimized nutrient utilization or rumen stability.

For acetate, propionate, isobutyrate, valerate, and hexanoate, we do not interpret isolated sampling-date contrasts as treatment-dependent temporal effects when the treatment × date interaction was nonsignificant. The observed omnibus time effects may reflect temporal adaptation or common dietary and environmental influences [35,36]. Mechanistic explanations involving specific microbial populations or fermentation pathways remain hypotheses because we did not measure rumen microbiota or fermentation flux.

Isovalerate concentrations differed between treatments and changed over time, while hexanoate showed an overall treatment effect. However, plasma concentrations cannot identify the ruminal source or specific microbial process responsible for these differences. Isovalerate can arise from branched-chain amino acid metabolism [37], and hexanoate has been associated with lipid-related fermentation pathways [38], but we did not measure rumen microbial composition, fermentation flux, or specific microbial populations. These observations should therefore be interpreted as systemic metabolite differences rather than direct evidence of altered proteolytic activity, microbial protein turnover, or lipid-derived rumen fermentation.

### 4.3. Effect of BP on Reproductive Parameters

Only a small proportion of females became pregnant (T-BP: 2/15; T-CTL: 3/15), limiting inference about reproductive performance. Plasma P4 was analyzed as a continuous endocrine variable and was not used to classify ovulation or luteal activity. Pregnancy status may have influenced later P4 concentrations. Reports from other species provide biological context for possible reproductive effects of BP [39,40], but the present data do not demonstrate improved ovarian function or reproductive performance in the goats studied.

We acknowledge that a limitation of this study is that the fixed 10 g/day dose (approximately 0.31 g/kg initial body weight/day) was not standardized to individual live weight and was selected without a pilot dose-titration study. Although BP has been associated with reproductive effects in monogastric species [39,40], extrapolation to seasonally breeding adult ruminants is uncertain. In our companion manuscript [21], putatively annotated metabolomic features related to tryptophan biosynthesis and modulating melatonin secretion warrants further experiments to confirm the mechanism [41]. These considerations, together with seasonal anestrus and the small number of pregnancies, limit generalizability. Future studies should evaluate weight-standardized dose ranges, longer exposure, multiple breeding seasons, and direct measurements of melatonin, ovarian activity, and steroidogenic function.

The companion manuscript [21] reported that metabolomic profiling identified treatment-group differentiation on days 21 (70 features with higher abundance and 16 with lower abundance) and 42 (52 with higher abundance and 17 with lower abundance) in T-BP compared to T-CTL, although metabolite identities remained putative. Pathway analyses associated several of these features with lipid- and cholesterol-related pathways relevant to steroid biology [42,43,44,45,46,47,48,49]. Additional differentially abundant features were annotated to prostaglandin and leukotriene pathways [50,51,52,53,54,55], which are implicated in ovulation, luteal function, implantation, inflammation, and vascular regulation, as well as endocannabinoid and retinoid pathways [56,57,58,59,60,61,62]. Observed differences in steroid conjugates, glucuronides, and sphingolipid-related features [63,64,65,66,67] further indicate potential alterations in steroid turnover and membrane signaling. While these annotations generate hypotheses for future targeted studies, our companion manuscript [21] does not confirm increased cholesterol availability, steroid synthesis, coordinated pathway regulation, gonadal function, or hormonal balance. Future research should evaluate functional and targeted validation to determine whether these metabolomic associations have physiological and reproductive significance.

### 4.4. Strengths and Limitations

This study used a controlled experimental design with consistent pen and management conditions and repeated sampling over time. Important limitations include use of plasma rather than ruminal SCFA measurements; absence of direct rumen fermentation or microbial measurements; incomplete characterization of the BP botanical, phenolic, and flavonoid composition; treatment diets that were not strictly isoproteic or isoenergetic; the fixed non-weight-standardized BP dose and absence of a pilot dose-titration study; only five pregnancies, which limited reproductive inference and may have influenced later P4 profiles; and incomplete archived documentation of covariance-structure selection and multiplicity adjustment for some repeated-measures contrasts. These limitations constrain mechanistic and reproductive interpretation and support cautious treatment of isolated sampling-date differences.

## 5. Conclusions

Bee pollen supplementation at 10 g/day for 6 weeks during the suboptimal breeding season did not produce consistent treatment effects on circulating glucose, insulin, IGF-1, or overall P4 concentrations. Most plasma SCFAs varied mainly over time; isolated date-specific contrasts were not interpreted as treatment-dependent effects when the treatment × date interaction was nonsignificant. Isovalerate showed a significant treatment × date interaction, whereas hexanoate differed overall between treatments without a significant interaction. Only five does became pregnant; therefore, fertility, prolificacy, and reproductive rate were summarized descriptively and do not support a reliable treatment comparison or improved reproductive performance. Under the conditions of this study, 10 g/day BP did not overcome seasonal reproductive inhibition. Future studies should use dose-ranging, direct rumen measurements, and appropriately powered reproductive designs.

## Figures and Tables

**Table 1:** Ingredient and nutrient composition of the diet offered during the experiment. The diet was calculated to meet the nutritional requirements for a 30-kg non-pregnant goat with limited physical activity [24].

Ingredient Composition in Diet (%)			
Bermuda hay		77	
Commercial Pellets (San Angelo AP40^®^)		15	
Cracked corn		8	
Nutrient Composition			
Parameter	Bermuda hay	Pellets AP40^®^	Bee Pollen
DM (%)	93.7	92	93
CP (%)	7.7	9	22.4
NeM (Mcal/kg)	0.58	*	1.16
TDN (%)	54	*	97.8
Calcium (%)	0.39	1.5	0.11
Phosphorus (%)	0.21	0.3	0.47

* Not reported by the company.

**Table 2:** Reported insulin and insulin-like growth factor 1 (IGF-1) values in goats supplemented with 10 g/day BP (T-BP) or carrier feed alone (T-CTL) during the sub-optimal breeding season. Interaction between Treatment and Time was analyzed as well (TRT × Time).

	Treatment							*p* Value	
	T-CTL	T-BP	T-CTL	T-BP	T-CTL	T-BP	SEM	TRT	TRT × Time
n	15	15	15	15	15	15			
	D −8		D 21		D 42				
Insulin (µIU/mL)	9.2	9.5	8.9	9.9	9.5	11.3	0.3	0.12	0.36
IGF-1 (ng/mL)	39.4	41.3	40.3	40.2	36.2	37.1	1.4	0.59	0.96

**Table 3:** Descriptive reproductive outcomes in goats supplemented with 10 g/day BP (T-BP) or carrier feed alone (T-CTL) during the suboptimal breeding season. We did not calculate inferential *p* values for these sparse reproductive outcomes.

Parameter	T-BP	T-CTL
n	15	15
Fertility (%)	13	20
Prolificacy (fetuses/pregnant doe)	1.5	1.0
Reproductive rate (%)	20.0	20.0

## Data Availability

The data presented in the manuscript were part of Adriana Rojas’s Thesis Project and are available from the corresponding author upon reasonable request. The plasma metabolomic dataset generated from the same experimental cohort was reported separately in the companion publication [21] and is not part of the analyses presented in this manuscript.
